# *Ghost Bird* – The Ivory-billed Woodpecker: Hopes, Dreams, and Reality

**DOI:** 10.1371/journal.pbio.1000459

**Published:** 2010-08-17

**Authors:** Jerome A. Jackson

**Affiliations:** Department of Marine and Ecological Sciences, Florida Gulf Coast University, Ft. Myers, Florida, United States of America

**Figure pbio-1000459-g001:**
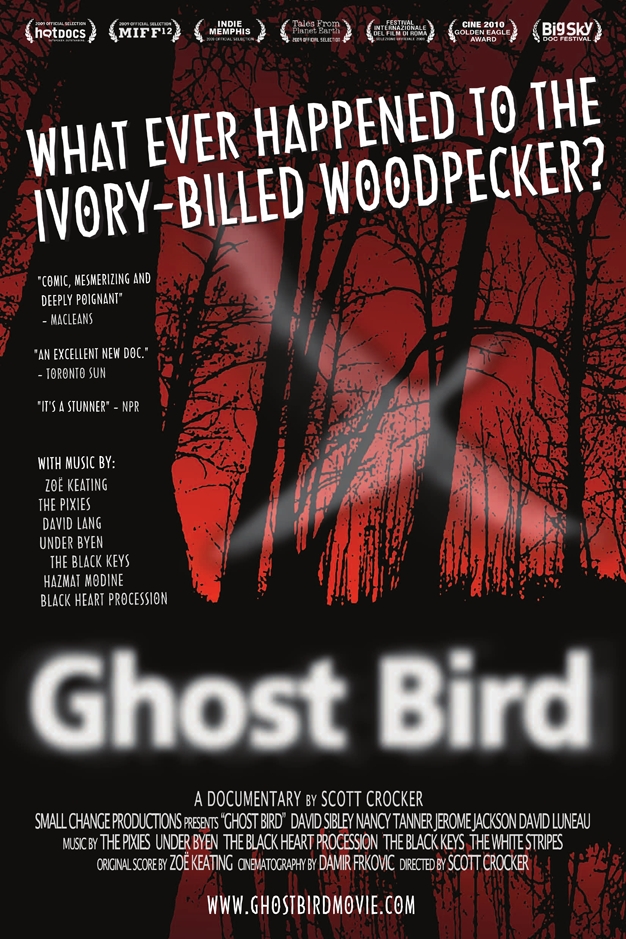
Scott Crocker (2009) *Ghost Bird.* Small Change Productions. www.ghostbirdmovie.com.

[Fig pbio-1000459-g001]Science is a human endeavor, replete with all the trappings and promise of science as well as all the foibles of humans. *Ghost Bird*, an 85-minute documentary film [1] about the possible rediscovery of the Ivory-billed Woodpecker (*Campephilus principalis*) in eastern Arkansas in 2004, bares the very fabric of science along with the diversity of public perceptions that color this fabric with hues of economic, political, and conservation hopes, dreams, and realities. This is a story about people perhaps even more than it is about the Ivory-billed Woodpecker. It is very much about the interfaces and interactions of science, conservation, culture, politics, and the news media.

I acknowledge up-front that I am interviewed in the film, served on the Ivory-billed Woodpecker Recovery Team of the US Fish and Wildlife Service, and was among the first to challenge the potential rediscovery of this iconic bird. I have been a lifelong student of woodpeckers and, in 2004, prior to knowledge of the rediscovery efforts, published a book detailing the history, behavioral ecology, and my own searches for the species [2]. One might say these facts introduce the potential for bias, but they also give me a unique perspective on the actual events as they unfolded, the film's faithfulness to the events, and the potential impacts of the film on science and the public perception of science.

At the scientific heart of the film is the claim by John Fitzpatrick and his colleagues of “confirmation” of the existence of the Ivory-bill in eastern Arkansas [3], and skepticism of that claim by others (e.g., [4],[5]). The claim was initially taken seriously because of several levels of “authority” that were evident: the credentials of the senior author, the large number of authors involved, the reputation of the Cornell Laboratory of Ornithology, the stature of the US Secretary of Interior (who announced the discovery), and the stature of the journal *Science*, in which the evidence was published. Crocker documents the “rediscovery” efforts, announcement, and claims through effective use of news video, since the Cornell Lab refused to allow their employees to be interviewed. Interviews with me, Richard Prum, and David Sibley highlight the counterclaim and interactions among the scientists, demonstrating the process of science as the search for firmer evidence proceeded without success. Prum, Sibley, and I were interviewed separately and each of us was unaware of what the others had said. Crocker edited these interviews, blending them to show the consensus among the skeptics and the details of arguments made. While *Ghost Bird* presents the story of a manuscript by the skeptics that Fitzpatrick convinced the authors to withdraw (from *PLoS Biology*), only two of the four authors of the manuscript are mentioned in the film (Prum and Jackson); the other authors were Mark Robbins and Brett Benz from the University of Kansas. We had all examined the *Science* article by Fitzpatrick and his colleagues, and they provided us a copy of the Luneau video—the single most important bit of data on which they based their paper. Based on the published paper and our analysis of the Luneau video, we concluded that the data were not strong enough to support the conclusion that the presence of Ivory-billed Woodpeckers in Arkansas had been confirmed.

The seeds for *Ghost Bird* dropped from the lush tangle of news stories on April 28, 2005, when the announcement was made by US Secretary of the Interior, Gale Norton, and Cornell Laboratory of Ornithology Director, John Fitzpatrick, that rediscovery of the Ivory-billed Woodpecker had been confirmed in eastern Arkansas along the Cache River just north of Brinkley, a town with fewer than 4,000 people. It was wonderful, positive news amidst a jungle of thorny negative stories in the preceding days and weeks. The news went global overnight and found fertile ground in the mind of Producer/Director Scott Crocker. Within a month, Crocker had contacted me, as well as others, as he began pursuing the story of this rediscovery. His search paralleled searches for the bird, taking him into the swamps, but also into the community of Brinkley, great museums and universities of North America, and deep into the history and lore of the Ivory-bill and the habitat it depended on.

Crocker began the film with no agenda other than documenting the rediscovery, the science, the roles and perceptions of those involved, and the impacts that rippled through eastern Arkansas and swirled through scientific, conservationist, and political communities. From his hours of diverse interviews, he wove a tale that accurately captures this complex backdrop, engages the audience, and flows smoothly.

**Figure 1:**
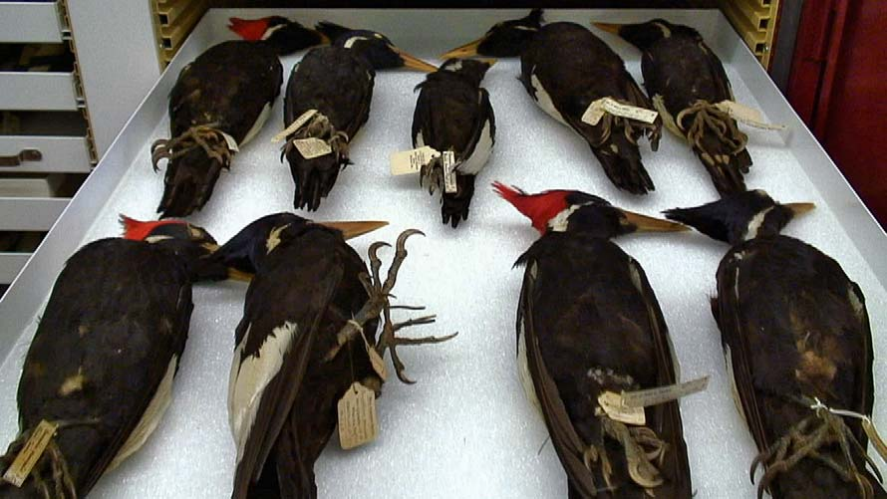
In a somber scene in *Ghost Bird*, workers at Harvard's Museum of Comparative Zoology close drawer after drawer of Ivory-billed Woodpecker specimens, still and lifeless, evoking the chilling finality of extinction. (Image: Damir Frkovic © 2009/Small Change Productions).

The tale of *Ghost Bird* is twice told—once by the words of those Crocker interviewed and again by director of photography Damir Frkovic. Camera angle and lighting—close-up or distant—all bring the viewer into each scene like the proverbial “fly on the wall.” Speeded up film of a caravan of cars and a flotilla of camouflaged searchers in kayaks headed for the swamp add a bit of humor, but more importantly insert the sense of frenzy that was truly there. Film of other media photographers in action, and interviewers plying searchers with questions enhance understanding of the magnitude of the story, the iconic nature of the Ivory-bill, and the idea that perhaps we still have a chance to change the trajectory of extinction and to redeem humanity for past losses. One dramatic scene, highly symbolic of the finality of extinction, is the silent footage of drawer after drawer of Ivory-billed Woodpecker specimens slowly being closed at Harvard's Museum of Comparative Zoology (see Figure 1).

Within *Ghost Bird* are multiple stories and perspectives skillfully interwoven to convey the complexity of the science, the public perception of science, the history of the landscape, the finality of extinction, the milieu of interactions, and impacts of each. At the cultural heart of the film is the story of how the potential rediscovery of the Ivory-bill, less than three miles from Brinkley, impacted the rural community. Before the discovery announcement, censuses had shown a declining population, boarded-up businesses were common, and only a local bank vice president, an avid birder, seemed to have heard of the Ivory-bill. Quickly, however, the town was on board the “Ivory-bill ship of salvation.”

Sales of camouflaged clothing soared. Demand for all things “Ivory-bill” led to the opening of an Ivory-billed Woodpecker store offering T-shirts, art prints, key chains, figurines, caps, postage stamps, and even an Ivory-bill-decorated toilet seat. The state boosted the excitement with a special Ivory-billed Woodpecker license plate and billboards boasting of the presence of the birds. A local motel changed its name to “Ivory-bill Inn,” Gene's Barbecue offered “Ivory-bill burgers,” and Penny's Haircare offered “Ivory-bill haircuts.” Almost all make cameo appearances in *Ghost Bird*. The mood of the community soared with dreams of renewal. Then hopes and dreams fell with news of skeptics and the lack of evidence to support Ivory-bill claims. Today, the gift shop is closed and the Ivory-bill Inn has reverted to its original name. The changing fortunes of the community are captured through a series of insightful and charming interviews with local citizens and symbolically depicted at the conclusion of the film by an ice-covered Ivory-billed Woodpecker sign and one lone searcher (me) standing in a snow storm under darkened skies at the Arkansas Highway 17 bridge, where so many had come to look for the Ivory-bill during warmer, brighter times.

“The Bird is the Word” became an unofficial slogan for Brinkley and appears in the movie on a sign in front of Gene's Barbecue, the local restaurant that became a meeting place for searchers and reporters. Taken from the 1963 hit song “Surfin' Bird” sung by a group known as The Trashmen, the catchy lyrics and hurried pace of the tune play a pivotal role in involving the audience with the film. Other music in the film also contributes significantly to the mood and tempo of the action.

While focusing on Harvard's collection of extinct North American birds, a strong and well-articulated message by Scott Edwards, curator of birds at the Museum of Comparative Zoology, identifies habitat destruction and over-hunting as causes of the extinction of the Carolina Parakeet (*Conuropsis carolinensis*), Passenger Pigeon (*Ectopistes migratorius*), Bachman's Warbler (*Vermivora bachmanii*), and potentially the Eskimo Curlew (*Numenius borealis*) and Ivory-billed Woodpecker. This section of the film invokes an emotional realization of all that we have lost and can never regain and engenders a spirit to preserve what we still have left.

Edwards notes that, more than for the other species, the extinction of the Ivory-billed Woodpecker may have been accelerated by collecting of scientific specimens. While this may be true, there are two important caveats that need to be considered: (1) cutting and severe fragmentation of the old growth forests almost certainly assured extinction of the Ivory-bill, and perhaps the Passenger Pigeon, with or without scientific collecting [6], and (2) laws did not protect these species during the 19th century, and collecting birds, their nests, and eggs was akin to collecting Beanie Babies or baseball cards during the late 20th century. Catalogs offered skins of Ivory-bills for sale, and entrepreneur hunters followed the loggers into the forest to collect the birds. Yes, more than a century ago scientists did collect some Ivory-bills, but institutions and government agencies were also involved in the collection of Ivory-billed Woodpeckers. Two of the last Ivory-bills known to have been collected were shot in 1924 in central Florida and sold to the Florida Museum of Natural History by two brothers with a permit from the state of Florida. The last I know of was shot by a Louisiana legislator to prove that the birds existed in the Singer Tract, a forest owned by the Singer Sewing Machine Company, near Tallulah, Louisiana, in 1932. He had been given a permit by the state so that he might prove their existence.

The collection of specimens, whether for science or for personal collections, probably represented a small fraction of those Ivory-bills killed by humans. They were a big target, considered edible, important symbols whose bills and scalps were used as decorations by Native Americans, and a source of fascination and awe because of their size and their ivory-colored bill [2],[7]. Many, perhaps most, of the slightly more than 400 specimens of Ivory-billed Woodpeckers in museums came from the private collections of individuals rather than through collection efforts by scientists. The web of complicity in the path to extinction for the Ivory-bill is broad and the tendrils of demise no doubt changed in their level of importance as habitats changed, human populations grew, and collecting of birds became a popular pastime.

James Tanner studied the Ivory-bills in the Singer Tract during the 1930s and monitored their decline during the early 1940s as the Chicago Mill and Lumber Company cut the forest [8]. His wife, Nancy, who accompanied her husband into the Louisiana Swamp in December 1941, adds grace, wisdom, and first-hand knowledge of living Ivory-bills to the film as she shares Tanner's photos, her own observations of the birds, and discusses the conversion of the forest to soybean fields.

Perhaps the last individual to document the Ivory-bill in North America was artist Donald Eckelberry who, in April 1944, was sent by the National Audubon Society to the Singer Tract to check on the status of the birds [9]. He found a lone female flying over the cutover forest, sketched it, and later painted the haunting scene. Eckelberry's sketches and painting and an interview with his wife Virginia poignantly document the weakened thread by which the fate of the species was hanging.

Throughout the film David Sibley, field-guide author, artist, and perhaps North America's best-known birder, weighs in with his own experience in the forest of eastern Arkansas and his evaluation of the evidence presented as “confirmation” of the existence of the Ivory-bill (see also [5]). Although Sibley is not an academically trained scientist, his reasoned position, understanding of bird behavior and ecology, and his stature within the birding community contributed greatly to public understanding of the scientific issues associated with the Ivory-billed Woodpecker story.

A new element at the interface between science and the public makes an important debut in *Ghost Bird*—the Internet blog. Tom Nelson, an electrical engineer from Minneapolis, Minnesota, began writing of his concerns about the quality of the evidence supporting rediscovery of the Ivory-billed Woodpecker shortly after the science was first questioned. Under the banner “Ivory-bill Skeptic” dozens of individuals contributed important discussion of the scientific issues. Nelson is interviewed in the movie and comments that it is “important to get the science right.”

David Sibley echoes Nelson's comments and touts the value of the blog in providing an anonymous outlet for those interested. Most of the comments were posted anonymously out of fear of being criticized by colleagues for taking one stance or another. Although it must be noted that anonymity also eliminates responsibility. Other blogs (such as Ivory-bills Live ???! and an adjunct to the WorldTwitch blog called Peckergate) also joined the discussions of how science was working or not working and might have been used. An issue that is prominently featured in *Ghost Bird* is the source of the 27 million or more federal dollars allocated to the Ivory-billed Woodpecker search and recovery efforts. Many conservationists were upset that very scarce funds might be sunk into the recovery of a species that doesn't exist, when other real, though rapidly declining endangered species go drastically underfunded. (Not to mention more ecosystem-based projects aiming to protect numerous species.) In response to queries regarding the funding, a federal official noted that in allocation of funds there are winners and losers.

Among the lessons of *Ghost Bird* are needs for the understanding of the roles and impacts of scientists and media in shaping the public perception of science. As a result of the initial press conference and announcement by the Secretary of the Interior, numerous press releases, and web pages by various scientists and organizations involved, the potential rediscovery of the Ivory-bill and the searches that ensued became very public. Scientists both used and were used by the media. Sound bites from scientists were readily picked up and became well known among the public. For example, use of the code name “Elvis” by the Cornell Laboratory of Ornithology to refer to the initial secret search for the Ivory-bill was revealed by the media and its meaning gradually morphed from “Elvis found” to “Elvis often seen but never confirmed” as sightings came in without evidence, akin to jokes that refer to “Elvis” having “entered the building.”

News reports by the media often included errors in fact and, through omissions, also led to false impressions. For example, in opening sequences of *Ghost Bird*, reporters in at least two of the news clips say that Ivory-billed Woodpeckers have been believed “extinct since the 1920s.” The truth is that in the 1920s the Ivory-bill *was* believed by some to have been extinct, but then it was rediscovered in 1924 and again in 1932, and persisted at least until 1944.

Often the media described individuals with incomplete or inaccurate titles that implied “authority,” enhancing the credibility of what they said. For example, the media often mentioned that some of the individuals involved were “professors” or “employees” at a college, university, or well-known institution without identifying their areas of expertise, when in fact their expertise has little or no relevance to the Ivory-billed Woodpecker. Some of this was error on the part of media, but some was in the form of news releases from individuals or institutions that provided unclear titles of some of those involved.

In *Ghost Bird*, aerial photographs clearly show the busy traffic along Arkansas Highway 17 and along Interstate 40 at the north and south ends of the approximately 3-mile stretch of the Cache River, buffered by an approximately one-mile–wide patch of forest, where the observations were made. As the news of the potential rediscovery of the Ivory-bill unfolded, an aerial photo of the area where the sightings occurred appeared on the Internet. It had been taken from an altitude such that the edges of the forest were clearly visible, showing that the forest was a ribbon of habitat bounded by cleared agricultural land. Within days, that photo was changed to one taken from a lower altitude, showing the big trees and no limits to the forest. This limitless forest impression was also conferred by a special CBS 60 Minutes report that referred to the area as the “Amazon of North America.”

Science is not a pristine enterprise where men or women in white coats offer edicts from on high, but rather a messy affair where findings are scrutinized and proof is demanded. At one point in the movie Richard Prum points out the fear that scientists might have of Rush Limbaugh making hay of the scientific quagmire that the Ivory-bill saga had become. Such public science is like that and sometimes results in scientists retreating into their labs and not dealing with media. But, as scientists, we need to be responsibly responsive to the public and the media in communicating not just the results but the practice of science.

*Ghost Bird* reveals this process and the myriad of impacts it can have. It is a film that will produce a more sophisticated citizen with a better understanding of how science works. While in many ways it is a fun film, a fascinating window on science and the interfaces of science, media, and the general public, ultimately, it tells the story of the tragic extinction of an iconic species and our collective and probably unfounded, yet seemingly inextinguishable hope that maybe, it might still exist. Science can prove that the Ivory-billed Woodpecker still flies. It cannot prove that it does not. With the efforts that have been made since 2004, it has become increasingly likely that it is extinct. But… the truth is still out there.

*Ghost Bird* will be screening theatrically in 20 cities throughout the US for one night only beginning this September. Screening information is available at www.ghostbirdmovie.com.
